# Need telomere maintenance? Call 911

**DOI:** 10.1186/1747-1028-2-3

**Published:** 2007-01-17

**Authors:** Sofia Francia, Robert S Weiss, Fabrizio d'Adda di Fagagna

**Affiliations:** 1 IFOM Foundation – FIRC Institute of Molecular Oncology Foundation, Milan, Italy; 2 Department of Biomedical Sciences, Cornell University, Ithaca, New York, USA

## Abstract

"Natura non facit saltum" (nature makes no leap) the Latins used to say, meaning that nature does not like discontinuities. Cells make no exception and indeed any discontinuity in the DNA double helix is promptly detected, triggering an alteration of cell proliferation and an attempt to repair. Yet, linear chromosomes bear DNA ends that are compatible with normal cell proliferation and they escape, under normal conditions, any repair. How telomeres, the chromosomes tips, achieve that is not fully understood. We recently observed that the Rad9/Hus1/Rad1 (911) complex, previously known for its functions in DNA metabolism and DNA damage responses, is constitutively associated with telomeres and plays an important role in their maintenance. Here, we summarize the available data and discuss the potential mechanisms of 911 action at telomeres.

## Background

Any discontinuity within the DNA double helix is perceived by the cell as a threat to its genetic integrity. Consequently, cells respond promptly to the generation of DNA interruptions by mounting a coordinated set of actions collectively known as the DNA-damage response (DDR) with the intent of arresting cell cycle progression and initiating DNA repair. In higher eukaryotes, the large protein kinases Atm and Atr play central roles in initiating the DDR [1]. Atm responds primarily to double-stranded DNA breaks (DSB). With assistance from the Mrell/Rad50/Nbsl complex, as well as additional mediator and adaptor proteins, Atm phosphorylates and thereby modulates the activity of several substrates involved in cell-cycle control and DNA replication. Bulky DNA lesions, processed DSB, and DNA replication blockage trigger a second DDR pathway involving Atr. These insults lead to the accumulation of single-stranded DNA (ssDNA) coated with the replication protein A (Rpa), a DNA structure that separately attracts Atr and a trimeric complex of Rad9, Rad1, and Hus1 (911) [2]. Atr, in association with its cofactor Atrip, is further stimulated through interactions with Topbpl and, with assistance from the 911 complex and Claspin, phosphorylates and activates the transducer kinase Chkl [3]. Chkl and other Atr targets then mediate a host of DDR outputs, including cell cycle arrest, replication fork stabilization, and DNA repair.

The 911 trimer resembles the toroidal sliding clamp proliferating cell nuclear antigen (PCNA) and is loaded onto DNA by a clamp loader composed of Rad17 and replication factor C (RFC) subunits [4]. While many DDR factors function exclusively in DNA damage signaling, the 911 complex also directly participates in DNA metabolism. The checkpoint clamp physically associates with several factors required for base excision repair (BER) [5-11] and additionally interacts with translesion DNA polymerases [12,13]. 911 function is also required for homologous recombinational repair (HR) [14], although the precise molecular details of this role have not been elucidated. These findings highlight a broad role for the 911 complex in a variety of DNA transactions and suggest a model in which this trimer may act as a scaffold to recruit checkpoint proteins and DNA modifying enzymes to their sites of action.

## Telomeres: an unexpected home for DDR factors

Telomeres, the ends of linear chromosomes, are exceptional, as they are DNA ends that do not normally trigger a robust DDR and are compatible with normal cellular proliferation in checkpoint proficient cells. They contain long stretches of DNA tandem repeats (TTAGGG in vertebrates) and terminate in a 3' protruding single-stranded DNA overhang. Due to the inability of the standard DNA replication machinery to fully replicate DNA ends, many cells maintain their telomeres by the action of telomerase (Tert), a specialized reverse transcriptase that uses its associated RNA component (Terc) as a template to elongate chromosome ends (see [15] for a historical perspective). In the absence of such a mechanism, some tumor cell lines maintain telomeres through homologous recombination, a mechanism known as ALT, for Alternative Lenghtening of the Telomeres [16]. The telomeric repeat sequences are recognized by a specific set of sequence- and structure-specific DNA-binding factors that are essential for many of the key biological features of telomeres, including their ability to avoid triggering a DDR. Some of these factors, such as Trf1 and Trf2, bind to the double-stranded portion of the telomeric DNA and are involved in telomere length regulation and protection, while others, such as Pot1 have important roles in capping the very end of the chromosome by virtue of their ability to recognize the telomeric 3' overhang [17-19]. In addition, the recently discovered KEOPS complex has also been shown to control telomere capping [20]. The intrinsic ability of telomeric tracts to prevent full DDR activation has been demonstrated in the yeast *Saccharomyces cerevisiae *in which an internal tract of telomeric repeats inhibits DDR signalling [21]. This anti-checkpoint function is likely dependent on telomere DNA binding proteins, and indeed in mammals inactivation of TRF2 triggers a robust DDR emanating from telomeres [22-24]; conversely, Trf2 overexpression can dampen DDR signalling, possibly by directly binding and inhibiting Atm [25].

Despite a tight inhibitory control of DDR at telomeres, mammalian chromosome ends are nevertheless a physiological substrate of the DDR apparatus as the inactivation of genes encoding certain DDR factors involved in DSB repair such as DNA-PK [26-30], Parp-1 [31,32], Rad51D [33] and Wrn [34], as well as components of the Atm-Mrell/Rad50/Nbsl pathway [35-38] results in telomere dysfunction. Thus, these DDR proteins, which ordinarily would vigorously oppose a free DNA end, actually serve to protect telomere integrity. Specifically how DDR factors are integrated with telomere physiology is, however, still largely unclear [19,39]. The observation that telomere DNA ends become transiently accessible in the G2 phase of the cell cycle [40] suggests that telomeres can engage DDR factors in a controlled and time-restricted fashion, without nevertheless enforcing a full blown cell-cycle checkpoint. It is therefore possible that telomeres are recognized only by the upstream elements of the DDR machinery, but do not engage the downstream elements of the cascade that would lead to cell-cycle checkpoint enforcement. It remains unclear at which step the transduction of the signal is inhibited. Overall, this strategy may allow DDR factors to mediate a subset of their functions at telomeres in order to promote telomere replication and stability, without triggering other unnecessary or even detrimental responses. These observations also raise the possibility that some DDR proteins might have special functions at telomeres in addition to their established activities in checkpoint signaling and DNA repair.

## 911, a new component of mammalian telomeres

Recently, we identified an important role for 911 at telomeres in mammals [41]. We reported that the 911 complex is physically associated with telomeric DNA in human and mouse cells. This association engages all three individual subunits of the complex and occurs both in normal and transformed cells, independent of telomerase expression or cell cycle stage. Therefore, 911 is a novel constitutive component of the mammalian telomere. In order to investigate the physiological significance of this association, we analyzed telomere length in cells bearing a genetic deletion of Hus1. Unfortunately, mouse telomere length cannot be reliably measured by Southern blotting techniques, because mouse telomeres are very long (10–50 Kb) and heterogeneous, bearing restriction enzymes cleavage sites interspersed within the telomeric tracts [42]. Techniques based on fluorescence in situ hybridization (FISH) are more reliable because they are insensitive to the polymorphisms associated with mouse telomeres and they can be used to specifically detect and measure only the telomeric repeats signal at chromosome ends [43]. By quantitative use of this technique, we demonstrated that Hus1 deficiency leads to dramatic telomere shortening, from a mean telomere length of 48 Kb in wild type fibroblasts to 27 Kb in Hus1-null fibroblasts. This result was supported by an independent analysis of telomere length by flow-FISH, another FISH-based technology, in unpassaged thymocytes from conditional Hus1 knockout mice. Therefore, 911 is associated with mammalian telomeres and the lack of one of its components, Hus1, leads to a dramatic telomere shortening. These data are in agreement with those showing telomere loss and chromosomal fusions in mouse and human cells lacking Rad9 [14]. Notably, the role for 911 at telomeres is evolutionarily conserved. *Caenorhabditis elegans *strains lacking HUS-1 or MRT-2, a functional ortholog of mammalian Rad1, display progressive telomere shortening and loss of germ-line immortality [44,45]. In *Schizosaccharomyces pombe *orthologues of this complex have been reported to associate with telomeric repeats and are required for telomere length maintenance [46]. The deletion of components of the orthologous complex in *Saccharomyces cerevisiae *causes mild telomere length changes, although some effects appear to be laboratory or strain specific [47-49].

## Possible mechanisms for 911 action at telomeres

A key question raised by the above-mentioned studies centres precisely on how the 911 complex participates in telomere maintenance. To determine whether 911 affected the function of telomerase, the enzyme that catalyzes telomere extension, we compared telomerase activity in wild type cells and cells lacking Hus1 or other 911 components. Excitingly, we discovered that lack of 911 leads to reduced telomerase activity without affecting the expression of the core components of the telomerase complex. We therefore conclude that 911 controls telomerase activity. The notion that 911 promotes telomerase activity is consistent with findings indicating that defects in 911 and Tert have equivalent and non-additive effects on telomere length in *C. elegans *[50]. Interestingly, other elements of the mammalian 911 signaling cascade such as Atr neither interacted with telomerase nor detectably affected telomerase activity *in vitro*. These results suggest a unique functional role for 911 in the regulation of telomerase activity. Meanwhile, additional DDR proteins further contribute to telomere homeostasis through other important functions such as promoting T-loop formation and suppressing chromosomal end joining as well as sister telomere exchanges [17,40].

The structural similarity that the 911 trimer shares with PCNA provides a useful starting point for considering how the 911 complex might influence telomerase activity. Best known for its role as a processivity factor for DNA polymerases during DNA synthesis, PCNA functions as a molecular scaffold that recruits replication and repair proteins to DNA [51]. Evidence suggests that the 911 complex might function in an analogous manner as a landing pad for replication, repair, and checkpoint proteins. For instance, the 911 complex has been shown to directly interact with two different polymerases involved in translesion DNA synthesis (TLS) Pol Kappa [13] and Pol Zeta [12]. Use of the 911 complex as an alternative clamp for damaged templates may provide a mechanism for controlling the usage of TLS polymerases, which have a low fidelity on undamaged DNA templates. Indeed, loss of MEC3 or DDC1, budding yeast orthologs of Hus1 and Rad9, respectively, results in decreased Pol Zeta-dependent spontaneous mutagenesis [12]. These results suggest that the 911 complex may directly control the access of Pol Zeta to damaged DNA, although stimulation of Pol Zeta-mediated translesion synthesis by the checkpoint clamp has not yet been recapitulated in vitro [52]. A scaffolding function also has been identified for the 911 complex during the process of base excision repair. 911 associates with proteins that act throughout the multi-step BER process, including the DNA glycosylase MutY homolog (Myh) [5,11], the flap endonuclease (Fenl) [7,10], DNA polymerase Beta [8], and DNA ligase I [6,9]. In some of these instances, 911 is known to directly stimulate the activity of the associated repair enzyme. Taken together, these findings establish a recurrent theme suggesting that a primary function of the 911 complex may be to recruit DNA modifying enzymes to appropriate substrates and to stimulate their activities.

When applied to the issue of telomere maintenance, this model would suggest that the 911 complex might act by directly binding and regulating the activity of telomerase. Consistent with this possibility, in a number of tests, 911 biochemically associates with catalytically active telomerase complex. The 911 complex is appropriately situated for such an interaction, as *in vitro *experiments suggest that 5'-recessed DNA ends are preferential sites for loading of the 911 complex, making telomeres a natural candidate substrate *in vivo *as well [53,54]. An association between 911 and telomerase could conceivably recruit telomerase to telomeres and/or enhance its activity. The latter could encompass improvements to telomerase processivity, similar to the effect of PCNA on DNA polymerase. However, it is worth noticing that the association of 911 with DNA polymerase Beta does not increase processivity but instead enhances primer utilization [8].

**Figure 1:**
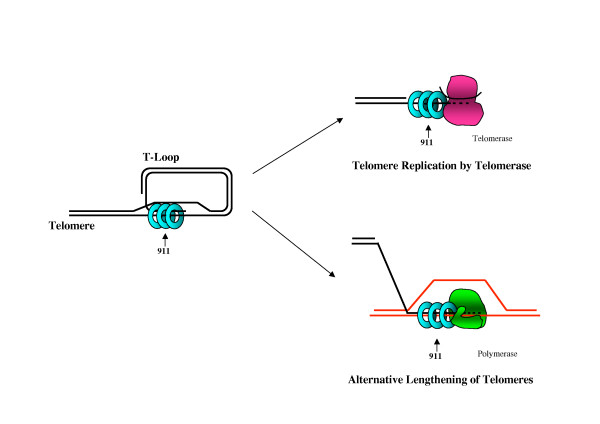
**Models for the function of the 911 complex at telomeres**. During most of the cell cycle, telomeres are believed to be folded into T-loop structures in which the 3' single-stranded overhang is tucked back into the adjacent double-stranded DNA, forming a D-loop. 911 is localized to telomeres in both quiescent and replicating cells, suggesting that it associates with T-loop structures. The precise DNA structure necessary for 911 loading at telomeres is unknown. In vitro studies suggest preferential loading of the 911 complex onto 5' recessed DNA ends, but loading onto 3' recessed DNA ends is observed under some conditions. The telomere shortening observed in 911-deficient cells as well as additional data implicate 911 in telomere replication mechanisms, including telomerase-based and alternative lengthening of telomeres (ALT) recombinational mechanisms. The speculative models shown illustrate direct interactions between 911 and telomerase or DNA polymerase, but the precise molecular function of 911 in these processes remains to be determined (see text for details).

Although the available data are consistent with a model in which 911 directly interacts with and stimulates telomerase (as shown in figure 1), it remains possible that 911 instead impacts telomerase through an indirect mechanism. 911 could fulfill such a role by helping create a telomeric substrate that is more accessible to telomerase or can be more readily extended. DNA polymerases are highly sensitive to misaligned primer/template substrates, terminal mismatches and other abnormal DNA structures. At telomeres, the G-rich single-stranded DNA overhang is prone to form G-quartet structures that are inhibitory for telomerase activity [55,56]. Optimal telomerase activity might require a mechanism for resolving such impediments at chromosome ends, a role that could be fulfilled by 911. In addition, the observation that DNA polymerase Beta localizes at telomeres [57] suggests a potential additional contribution of 911 to telomeric DNA replication.

Notably, the rate of telomere shortening that we observe in Hus1-null cells is higher than that reported for mice lacking telomerase, suggesting that additional mechanisms of telomere shortening might be at play in the absence of the 911 complex. Rapid telomere shortening could be caused by nucleolytic degradation or aberrant recombination events at uncapped telomeres. The 911 complex has been implicated previously in telomeric recombination. In ALT cells, telomeres are maintained by a recombinational mechanism in which short telomeres invade homologous telomeric sequences and use this as a template for lengthening [58]. Interestingly, ALT cells have been shown to display cytologically detectable accumulation of 911 at telomeres [59], suggesting that, in the absence of telomerase, the presence of the 911 complex at telomeres could promote telomere lengthening through HR. Moreover, Rad9 or Hus1 inactivation in mammals is associated with defective HR [6,14], which in some cases might result in increased usage of alternative, error-prone mechanisms. Thus, aberrant recombination events at telomeres could contribute to rapid telomere shortening in cells lacking the 911 complex.

Finally, it is worth considering that the presence of a poorly functioning telomerase on DNA might be more detrimental to telomere stability than not having telomerase at all. A poorly processive polymerase on DNA can cause DNA replication fork stalling and such an event in checkpoint-deficient cells has been shown to lead to fork reversal and hyper-recombination [60]. In agreement with this, budding yeast cells replicating with a limited amount of DNA polymerase show chromosomal instability at specific genomic regions [61]. Stalled forks promote checkpoint activation by exposing significant amounts of single-stranded DNA (ssDNA) coated by Rpa [60,62]. These Rpa-ssDNA complexes recruit Atr and 911 to stalled forks and cause the activation of replication checkpoints [63]. In checkpoint mutants, stalled forks rapidly degenerate, accumulating gapped and hemireplicated molecules that often end up processed by unscheduled recombinogenic events [64]. Therefore, it is possible that in cells lacking Hus1 the presence at the telomere of a poorly functioning telomerase complex leads to generation of aberrant DNA structures that can result in the rapid loss of telomeric repeats through low fidelity DNA recombination and processing events.

## Summary

The linear structure of eukaryotic chromosomes creates a requirement for special mechanisms to replicate and protect chromosome ends. Over the past several years, a seemingly unlikely caretaker of telomeres has emerged. DDR proteins, which normally act to eradicate free DNA ends, have proven to be critical to telomere homeostasis. As reviewed here, the 911 complex is the latest example of a mammalian DDR factor that localizes to telomeres and acts to maintain telomere integrity. Consistent with results from *S. pombe *and *C. elegans*, loss of 911 components leads to telomere shortening and end-to-end chromosome fusions. Moreover, the 911 complex can be co-immunoprecipitated with telomerase activity and is required for optimal telomerase activity in *in vitro *assays. The precise molecular function of 911 at telomeres, which may involve stimulation of telomerase directly through a physical interaction or indirectly through effects on the telomeric DNA substrate, has yet to be completely resolved. This and other aspects of telomere maintenance undoubtedly will continue to be areas of intense interest given the principal role of telomeres as determinants of cellular lifespan and genome stability.
